# Evaluation of indices for the assessment and classification of keratoconus based on optical coherence tomography and Scheimpflug technology

**DOI:** 10.1111/opo.13425

**Published:** 2024-12-05

**Authors:** Robert Herber, Janine Lenk, Lisa Ramm, Dierk Wittig, Maria Magdalena Patzner, Lutz E. Pillunat, Frederik Raiskup

**Affiliations:** ^1^ Department of Ophthalmology University Hospital Carl Gustav Carus, TU Dresden Dresden Germany

**Keywords:** ANTERION, corneal tomography, epithelium thickness, keratoconus, Pentacam, swept‐source optical coherence tomography

## Abstract

**Purpose:**

To compare the parameters and indices of a novel swept‐source optical coherence tomography device (SS‐OCT, ANTERION) with those of a rotating Scheimpflug camera (RSC)‐based tomograph (Pentacam) in normal and keratoconic (KC) eyes.

**Methods:**

This prospective, monocentric, cross‐sectional study included individuals with unoperated normal and KC eyes, selecting one eye per subject. Ectasia‐specific parameters analysed with the SS‐OCT were difference in mean keratometry (*K*_mean_) in the inferior and superior meridians, maximum keratometry value (*K*_max_), elevation of the posterior surface at the thinnest point, screening corneal objective risk of ectasia (SCORE) and thinnest point thickness. With the RSC, parameters determined were Belin/Ambrosio total deviation value (BAD‐D), index of height decentration and index of vertical asymmetry. KC classification with the SS‐OCT was based on the anterior and posterior radii of curvature and thinnest point thickness according to the ABCD classification of the RSC system.

**Results:**

This study included 117 individuals with healthy eyes and 335 eyes with KC. The indices with the highest diagnostic discriminatory ability between the two cohorts were SCORE, difference of *K*_mean_ in the inferior and superior meridians and posterior elevation of the thinnest point (SS‐OCT), as well as the index of height decentration, index of vertical asymmetry and BAD‐D (RSC). The classifications using SS‐OCT defined mild‐stage KC as *K*_max_, posterior elevation of the thinnest point and thinnest point thickness as ≤50.9 D, ≤30 and ≥472 μm, respectively. Moderate stage values were 51–55.9 D, 31–69 and 471–438 μm, respectively, while respective advanced stage were ≥56 D, ≥70 and ≤437 μm.

**Conclusion:**

The diagnostic capabilities for both devices were found to be comparable. KC classification using SS‐OCT can be independently based on the anterior surface, posterior surface and corneal thickness.

**Key points**

- Swept‐source‐based optical coherence tomography is a new procedure in ocular anterior segment diagnostics that enables precise measurements of corneal tomography.
- The main parameters of the swept‐source‐based optical coherence tomography for screening keratoconus correspond with those of the Scheimpflug‐based tomograph.
- Staging into mild, moderate and advanced keratoconus can be made using the parameters maximum keratometry, the posterior elevation of the thinnest point and the thinnest point thickness with the swept‐source‐based optical coherence tomograph.

## INTRODUCTION

In recent years, corneal imaging has advanced rapidly due to technological advances in measurement devices and surgical procedures, including refractive surgery. This progress has been driven, not only by enhanced imaging, but also by the ability to quantify data through techniques such as topography and tomography. Computer‐assisted videokeratoscopy using a Placido disk has been a commonly used method for evaluating corneal topography. The disadvantage is the lack of information about the geometry of the posterior corneal surface and corneal thickness distribution.^1^, ^2^ The introduction of rotating Scheimpflug camera‐based tomography (RSC), a crucial development, allowed tomographic visualisation of the cornea, thereby providing data on both the anterior and posterior corneal surfaces as well as corneal thickness.^1^ Currently, tomographic and biomechanical assessments of the cornea are indispensable for the screening and management of corneal ectasia, especially keratoconus (KC).^3^, ^4^, ^5^, ^6^ KC, which develops primarily in adolescence and young adulthood, is a corneal disease characterised by the steepening and thinning of the corneal tissue, followed by loss of visual acuity. Owing to the progressive nature of the disease, regular monitoring (tomographic assessment) is essential to detect disease progression and initiate appropriate treatment. In recent decades, corneal cross‐linking has become the therapeutic gold standard, effectively stiffening tissue and stabilising the disease over the long term.^7^, ^8^, ^9^

Scheimpflug‐based imaging devices are widely available for tomographic assessment. These include the Pentacam HR (Oculus Optikgeräte GmbH, oculus.de/), which quantifies several parameters and indices suitable for detecting corneal ectasia. The ability to differentiate between healthy and keratoconic eyes has been demonstrated in numerous studies.^10^, ^11^, ^12^, ^13^, ^14^, ^15^ Optical coherence tomography (OCT) imaging of the anterior segment of the eye is possible using both high‐resolution spectral‐domain OCT with a wavelength of 845 nm and swept‐source OCT (SS‐OCT) with a wavelength of 1300 nm. This enables detailed quantification of corneal parameters, such as anterior and posterior curvature radii and corneal thickness. The ANTERION SS‐OCT (Heidelberg Engineering GmbH, heidelbergengineering.com/de/) is a novel device that enables tomographic and biometric measurements of the cornea or eye. Two previous studies have shown that SS‐OCT is highly comparable with Scheimpflug‐based tomography in both healthy eyes and those with KC; however, alternating between the two should be avoided.^16^, ^17^ Moreover, SS‐OCT allows a special view that enables both screening and monitoring of corneal ectasia and analysis of epithelial thickness with high repeatability.^18^ This overall index, known as Screening Corneal Objective Risk of Ectasia (SCORE),^19^ and the individual parameters from the “radar map” were evaluated in this study with respect to their suitability for assessing the severity of KC. Furthermore, the diagnostic ability of SS‐OCT to detect KC was compared with that of the RSC.

## METHODS

The protocol for this prospective, single‐centre, observational study was reviewed and approved by the Ethics Committee in accordance with the principles of the Declaration of Helsinki. The trial is registered on Clinicaltrials.gov under the number NCT04251143. Participants were recruited from the KC and refractive consultation centre between 2019 and 2023 and were required to provide informed consent. All participants underwent a comprehensive ophthalmological examination, which included a medical history assessment using a questionnaire specifically designed for KC, as well as an anterior and posterior segment slit‐lamp examination to evaluate endothelial status, visual acuity and refraction.

Inclusion criteria for individuals with healthy eyes were normal topography and tomography measured by RSC (with maximum keratometry values [*K*_max_] <47 D, an inferior–superior difference <1.5 D and a skewed radial axis index <22°), as well as the absence of abnormal signs in anterior and posterior elevation maps, corneal thickness distribution and corneal scars. KC diagnosis was made by topography and tomography (central or inferior steepening of the corneal radii) and pachymetry maps (thinning of the cornea) using the “4‐refractive map display” of the RSC. Clinical signs, such as Vogt's striae or Fleischer's ring were also documented. Only participants with clinical KC in both eyes were included. Before the examination, the participants avoided wearing contact lenses for 14 days. The exclusion criteria were previously operated eyes or other corneal ectasias, such as pellucid marginal degeneration and keratoglobus, endothelial guttae or other ophthalmological diseases. Only one eye of each subject was included in the statistical analysis. One eye was selected at random if both eyes met the inclusion criteria.

### Scheimpflug‐based tomography

A Scheimpflug tomography system (Pentacam HR, Oculus Optikgeraete GmbH) was used. This tomographer measured the anterior segment of the eye using a rotating Scheimpflug camera, which was illuminated with a blue light slit during the measurement process. Various parameters were then derived using a three‐dimensional reconstruction of the cornea. The following topographical indices and parameters were selected for evaluation in this study: central keratoconus index, keratoconus index, keratoconus percentage index (KISA) index, maximum keratometry (*K*_max_), height asymmetry index, height decentration index, surface variance index and vertical asymmetry index. The selected pachymetry parameters were the minimum corneal thickness, average pachymetric progression and Ambrosio relational thickness. Furthermore, elevation data of the posterior corneal surface at the thinnest point of the cornea and the Belin/Ambrosio total value (BAD‐D) were analysed.

The ABCD grading system, developed by Belin and Duncan^20^ and adapted from the Amsler‐Krumeich classification, was used to classify KC. The cornea was classified based on individual criteria, such as the anterior corneal surface (parameter A), posterior corneal surface (parameter B) and corneal thickness (parameter C). The staging of KC was based on the average radius of curvature of the anterior corneal surface and the posterior corneal surface of a 3‐mm zone in the thinnest part of the cornea (corresponding to the centre of this zone). In addition, corneal thickness was measured in the thinnest area. Furthermore, it was possible to include the participant's visual acuity (D parameter),^20^ although this was not considered here owing to inconsistencies in the visual acuity data (visual acuity without or with contract lens correction or with glasses). Therefore, this classification is referred to as the ABC classification.

### Swept‐source OCT‐based tomography

Swept‐source‐based anterior segment OCT (SS‐OCT; ANTERION, Heidelberg Engineering GmbH) captures images of the anterior segment of the eye using 65 radial B‐scans, comprising 256 A‐scans, at a scan speed of 50,000 A‐scans/s. This process generates 16,640 measurement points, from which the tomographic data are derived. An active eye tracker enables the scan to be centred on the corneal vertex.

**FIGURE 1:**
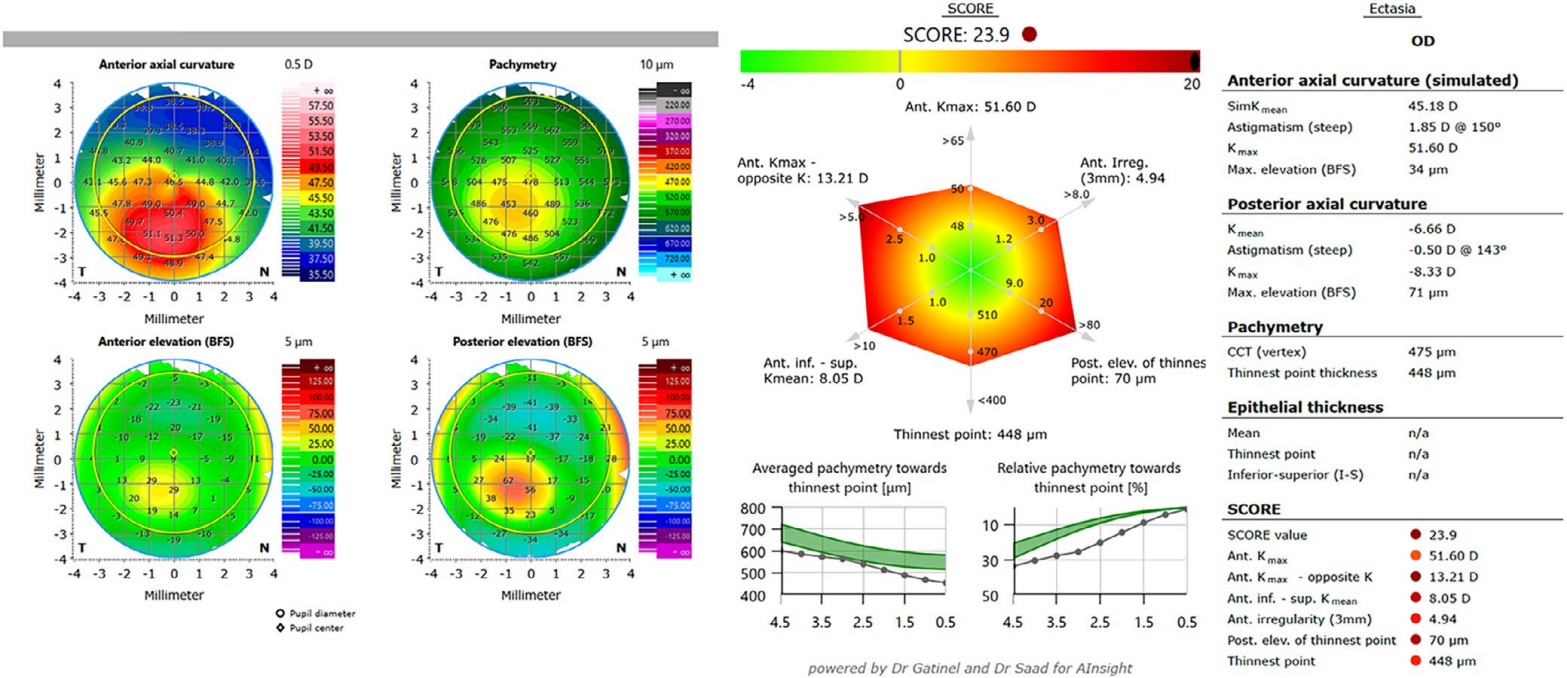
‘Ectasia’ view of the swept‐source optical coherence tomography (SS‐OCT) device with axial curvature map of the anterior corneal surface, corneal thickness map, elevation map of the anterior and posterior surfaces (left), Screening Corneal Objective Risk of Ectasia (SCORE) value (top right), radar map (centre right) and corneal thickness distribution (bottom right).

By default, the ectasia view (‘Ectasia’ in the ‘Cornea App’, Figure 1) displays four relevant corneal tomography maps: the axial corneal curvature of the anterior surface, corneal thickness map and elevation maps of the anterior and posterior surfaces. The SCORE parameter is indicated on a colour scale ranging from −4 to 20. Eyes with a low risk of ectasia have values <0, placing them in the green range. In contrast, eyes with a higher risk have values >0 and fall into the yellow, orange or red range, depending on the severity. The index combines corneal parameters from both the front and back surfaces.^19^ The ‘Radar Map’ comprises six additional individual parameters, which include the corneal curvature of the anterior surface, elevation data of the posterior surface and corneal thickness (thinnest part of the cornea). The corneal thickness distribution from the thinnest point to the periphery in the ring zones and the percentage increase in thickness are also shown in Figure 1.

**FIGURE 2:**
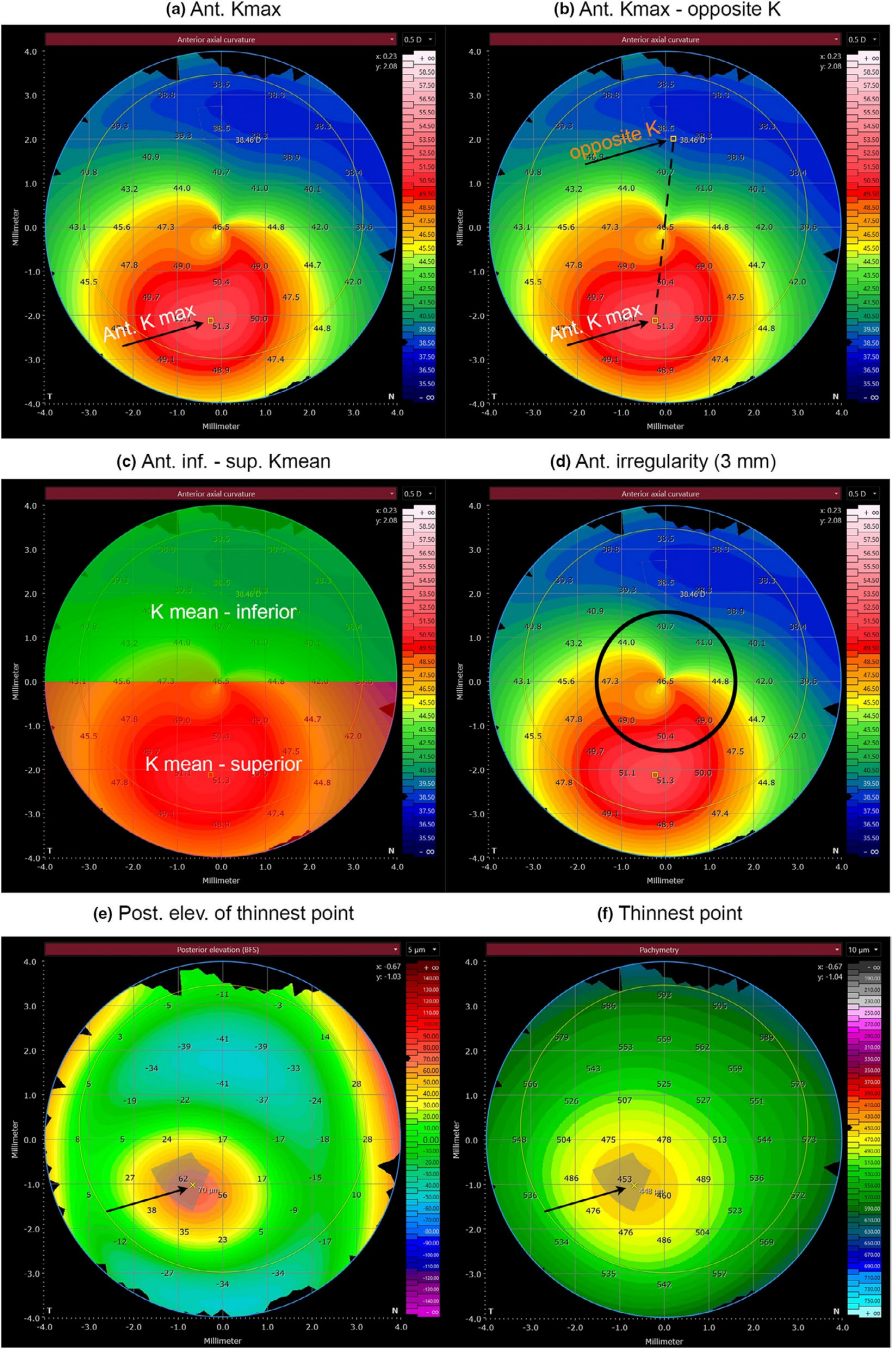
Overview of the radar map parameters. (a) *K*_max_: maximum keratometry value of the anterior surface; (b) *K*_max_—opposite *K*: difference of the maximum keratometry value and the opposite keratometry value; (c) Inf–sup *K* mean: difference of the mean keratometry value below (inferior) and above (superior) the merdian; (d) anterior irregularity (3 mm): irregularity of the axial curvature in the central 3 mm ring; (e) posterior elev. of the thinnest point: posterior elevation at the thinnest point of the cornea; (f) thinnest point: thinnest point of the cornea.

The six individual parameters of the radar map were as follows (Figure 2): the maximum keratometry value of the anterior corneal surface (*K*_max_ [D]), difference between the maximum keratometry value and the opposite keratometry value of the anterior corneal surface (*K*_max_ − opposite *K* [D]), difference between the mean inferior curvature and the mean superior value of the corneal curvature (inferior–superior *K* mean [D]), irregularity of the axial curvature in the central 3‐mm ring (anterior irregularity [3 mm]), posterior elevation (best‐fit sphere as the reference surface) at the thinnest point of the cornea (posterior elevation of thinnest point [μm]) and the thinnest point of the cornea (thinnest point [μm]).

Additionally, SS‐OCT measures the epithelial thickness distribution, where the mean epithelial thickness was analysed in the 2‐, 4‐ and 6‐mm zones. The standard deviation of the epithelial thickness (epithelium thickness std) describes the deviation over the entire 7‐mm zone. Moreover, the inferior–superior difference is defined by the manufacturer as the ‘difference between the mean thickness in the inferior region and the mean thickness in the superior region of the epithelium within the 0–7 mm zone’.

The measurements were conducted under consistent conditions, with reduced room lighting, although this was not required for SS‐OCT measurements. Participants were asked to blink before the measurement and then look at the fixation light. Only measurements with sufficiently good quality were used (Scheimpflug system: ‘ok’, measurements with ‘model’ were also accepted if the individual Scheimpflug images were of good quality; SS‐OCT: ‘pass’ and ‘borderline’).

### Statistical analysis

Statistical analyses were performed using the MedCalc software (MedCalc Software Ltd., medcalc.org/). Metric data were expressed as mean ± standard deviation (95% confidence interval) or median and interquartile range (IQR). The Shapiro–Wilk test was used to test for normal distribution of the data. Subsequently, normally distributed data were tested using the unpaired *t*‐test and non‐normally distributed data were tested using the Mann–Whitney *U* test. The χ^2^‐test was used for the statistical testing of categorical data. To analyse the agreement between the measuring devices, Bland–Altman diagrams were created and the mean offset and limits of agreement (LoA) were determined. Moreover, the association between the size of the values and differences between the devices were evaluated using regression analysis, which determined the slope (beta value) of the linear equation (pink line in Data S2). Pearson's correlation coefficient was used for correlation analysis. To determine the cut‐off values for the KC classification of the SS‐OCT device, the receiver operating characteristic (ROC) curve was used and its sensitivity and specificity were determined using the Youden index.^21^ In addition, the DeLong test was used to analyse the values of the area under the curve (AUC) for certain parameters with regard to diagnostic capability.^22^ Statistical significance was set at *p* < 0.05.

## RESULTS

### Demographic data

In this study, we included 117 eyes from 117 healthy participants and 335 eyes from 335 participants with KC. The mean age (95% CI) was 31.3 ± 10.1 (29.4–33.1) years in the healthy group and 34.8 ± 10 (33.8–35.9) years in the KC group. This difference was statistically significant (*p* = 0.001), but not clinically relevant. The sex distribution in the healthy group was 50%, whereas significantly more male participants (70%) were found in the KC group (*p* < 0.001). There were no significant differences in the laterality of the eyes (healthy group: right eye, 47%; KC group: right eye, 50%; *p* = 0.66). According to the ABC classification, the eyes in the KC group were classified for the parameter A (the anterior corneal surface) as follows: grade 0, 20.3%; grade 1, 15.8%; grade 2, 35.2%; grade 3, 9.3% and grade 4, 19.4%. For the parameter B (posterior corneal surface), the frequencies of the respective severity levels (from 0 to 4) were 5.7%, 6.5%, 36.7%, 10.7% and 40.3%. The distribution of stages for the parameter C (thinnest point thickness) was (from 0 to 4): 20.9%, 23.3%, 29.3%, 20.9% and 5.7%, respectively.

### Tomographic and agreement analysis of RSC and SS‐OCT

Table 1 presents the values of the RSC tomography parameters for the healthy and KC eyes. Statistically significant differences were found between the groups for all parameters (all *p* < 0.001). The results were similar for SS‐OCT parameters (all *p* < 0.001; Table 2). In Data S1, the Bland–Altman analysis demonstrates significant differences between RSC and SS‐OCT for steep and maximum keratometry with a large LoA of ~1 D in healthy eyes (*p* < 0.001). No significant relationship was found between the magnitudes of the values (no significant slope in the regression analysis, steep keratometry: *p* = 0.80, maximum keratometry: *p* = 0.93). In contrast, steep and maximum keratometry and corneal astigmatism differed significantly between RSC and SS‐OCT in KC with LoA of 3.29 D (steep keratometry), 3.72 D (corneal astigmatism) and 6.15 (maximum keratometry). There was a significant relationship between the size of the values and the differences between the devices (significant slope in the regression analysis, steep keratometry: *p* < 0.001, maximum keratometry: *p* < 0.001). For example, the higher the steep and maximum keratometry readings, the greater the difference between RSC and SS‐OCT, which correspond with higher severity. This indicates that depending on increasing KC stages, RSC measures higher steeper and maximum keratometry values than the SS‐OCT. For the posterior surface, the best‐fit sphere and elevation at the thinnest point were evaluated. The best‐fit sphere differed significantly between the devices in healthy and KC eyes. However, no association was noted between the magnitudes of these values. The mean offset of the elevation at the thinnest point was 0.7 μm (*p* = 0.02) with a LoA of 12 μm for healthy eyes and no relationship was found between the magnitude of the values (*p* = 0.83). In contrast, the mean offset in KC was −13.3 μm with LoA from −43.0 to 16.5 (*p* < 0.001). A strong association was found between the magnitude of the elevation value and the differences between RSC and SS‐OCT (*p* < 0.001), with the measured elevation values for SS‐OCT being higher than that for RSC, starting from an elevation value of 50 μm at the thinnest location.

**TABLE 1:** Overview of the indices of the rotating Scheimpflug camera tomography for the healthy and keratoconus groups.

Parameter	Healthy				Keratoconus				*p*‐Value
	Mean	SD	95% CI	Median (95% CI)	Mean	SD	95% CI	Median (95% CI)	
ISV	20.6	7.6	19.2–22.0	20 (18–21)	84.4	37.3	80.4–88.4	77 (71–86)	<0.001^a^
IHA	6.0	5.2	5.0–6.9	4.3 (3.5–5.3)	32.2	24.8	29.5–34.8	25.8 (22.4–28)	<0.001^a^
IVA	0.123	0.053	0.113–0.133	0.11 (0.1–0.13)	0.920	0.436	0.873–0.967	0.87 (0.8–0.94)	<0.001^a^
IHD	0.010	0.006	0.009–0.011	0.01 (0.009–0.01)	0.127	0.068	0.120–0.134	0.114 (0.105–0.128)	<0.001^a^
KI	1.013	0.021	1.010–1.017	1.01 (1.01–1.016)	1.232	0.124	1.218–1.245	1.21 (1.19–1.23)	<0.001^a^
CKI	1.007	0.005	1.006–1.008	1.01 (1.01–1.01)	1.056	0.056	1.050–1.062	1.04 (1.04–1.05)	<0.001^a^
*K*_max_ (D)	44.5	1.5	44.3–44.8	44.7 (44.2–45.1)	54.9	6.8	54.1–55.6	53.4 (52.5–54.2)	<0.001^a^
KISA	6.5	8.8	4.9–8.1	3.5 (3.0–4.2)	1934.0	3613.8	1545.6–2322.4	421.8 (348–481)	<0.001^a^
BAD‐D	0.98	0.53	0.88–1.07	0.99 (0.84–1.18)	8.27	4.06	7.83–8.7	7.45 (6.75–7.99)	<0.001^b^
RPI Avg.	1.002	0.098	0.984–1.020	1.0 (1.0–1.0)	2.011	0.705	1.935–2.087	1.85 (1.77–1.94)	<0.001^a^
ART Max.	447.4	67.3	435.1–459.7	444.0 (430.0–461.6)	178.3	75.0	170.2–186.4	174 (166–179)	<0.001^a^
Ele B thinnest point (μm)	6.7	3.6	6.0–7.3	6.0 (6.0–7.0)	48.8	22.7	46.4–51.3	46 (42.13–48)	<0.001^a^
MCT (μm)	547	33	541–553	547 (537–556)	465	41	460–469	465 (460–470)	<0.001^b^

Abbreviations: ART max, Ambrosio relationed thickness; BAD‐D, Belin/Ambrosio total deviation value; CI, confidence interval; CKI, Centre keratoconus index; Ele B, elevation data of the posterior corneal surface at the thinnest corneal thickness; IHA, Index of height asymmetry; IHD, Index of height decentration; ISV, Index of surface variance; IVA, Index of vertical asymmetry; *K*_max_, maximum keratometry value; KI, Keratoconus index; KISA, keratoconus percentage index; MCT, minimal corneal thickness; RPI Avg, averaged pachymetric progression; SD, standard deviation.

^a^ Mann–Witney *U* test.

^b^ Unpaired *t*‐test.

**TABLE 2:** Overview of the indices of the swept‐source optical coherence tomography for the healthy and keratoconus groups.

Parameter	Healthy				Keratoconus				*p*‐Value
	Mean	SD	95% CI	Median (95% CI)	Mean	SD	95% CI	Median (95% CI)	
Ant. *K*_max_ (D)	44.7	1.5	44.5 to 45.0	44.8 (44.5 to 45.2)	54.0	6.1	53.4–54.7	52.6 (51.9–53.5)	<0.001^a^
Ant. *K*_max_ − opposite *K* (D)	0.7	0.62	0.59 to 0.82	0.52 (0.44 to 0.64)	8.6	5.9	8.0–9.2	7.8 (7.0–8.8)	<0.001^a^
Ant. inf. − sup. *K*_mean_ (D)	−0.12	0.48	−0.2 to (−0.03)	−0.09 (−0.2 to 0.02)	6.12	4.15	5.68–6.57	5.42 (4.85–5.87)	<0.001^a^
Ant. irregularity (3 mm)	0.92	0.57	0.82 to 1.03	0.75 (0.62 to 0.91)	3.52	1.61	3.34–3.69	3.21 (3.04–3.41)	<0.001^a^
Post. elev. of thinnest point (μm)	6.0	4.0	5.0 to 7.0	6.0 (5.0 to 6.0)	61.9	31.4	58.5–65.3	57.0 (51.0–61.9)	<0.001^a^
SCORE	−1.5	0.83	−1.65 to (−1.34)	−1.6 (−1.81 to −1.40)	18.3	11.1	17.1–19.5	16.2 (14.8–17.8)	<0.001^a^
Thinnest point thickness (μm)	546	34	540 to 553	546 (540 to 556)	466	42	462–471	468 (461–473)	<0.001^b^

Abbreviations: Ant, anterior; CI, confidence interval; elev, elevation; inf, inferior; *K*, keratometry; *K*_max_, maximum keratometry value; *K*_mean_, mean keratometry value; Post. elev. of thinnest point, elevation data of the posterior corneal surface at the thinnest corneal thickness; SCORE, Screening Corneal Objective Risk of Ectasia; SD, standard deviation; sup, superior.

^a^ Mann–Witney *U* test.

^b^ Unpaired *t*‐test.

### Classification of tomographic parameters of the SS‐OCT in relation to RSC

**FIGURE 3:**
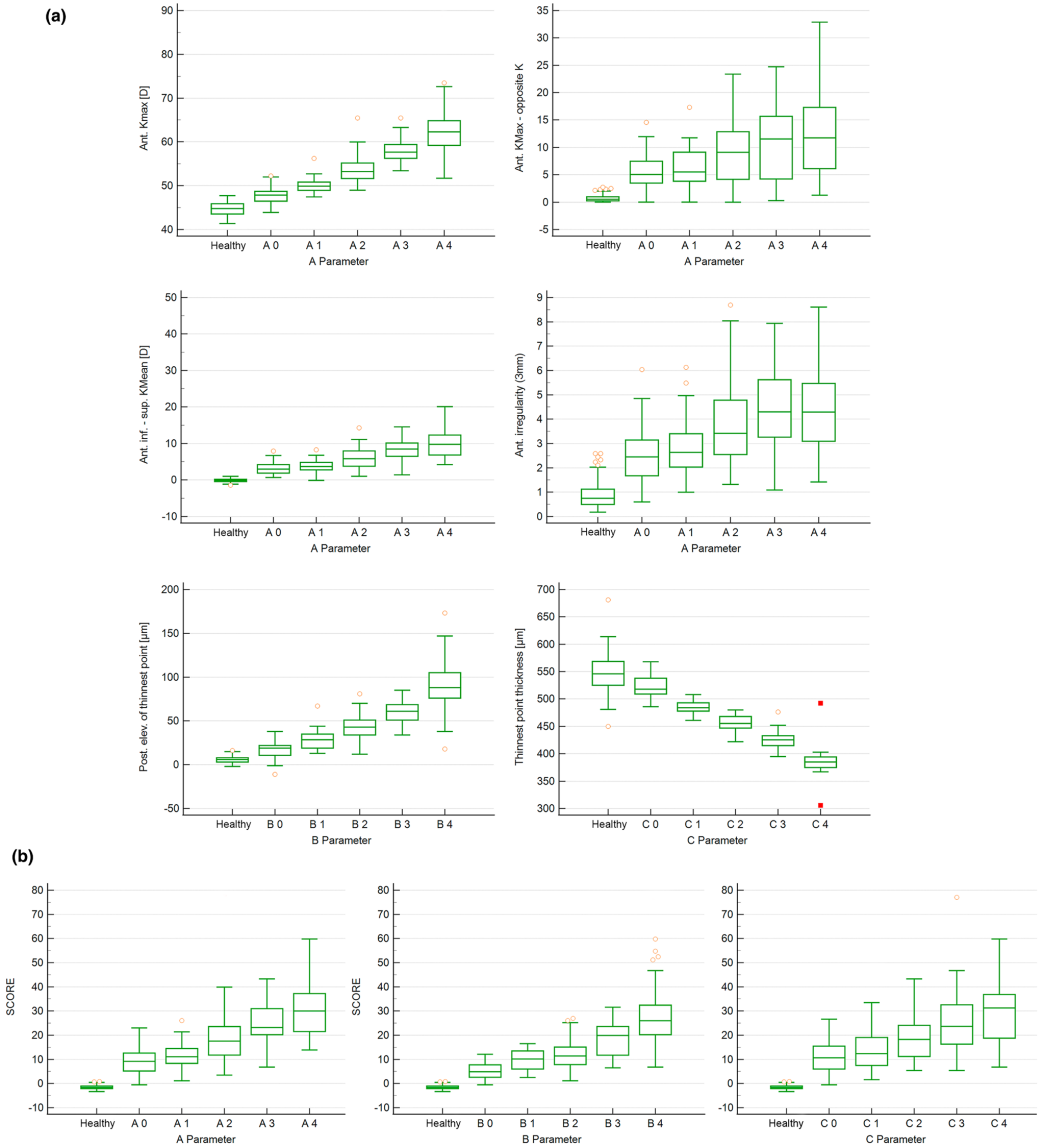
(a) Box‐plot of the single parameters of the swept‐source optical coherence tomography (SS‐OCT) depending on the ABC classification. Ant, anterior; A parameter, staging of the anterior corneal surface from 0 to 4; B‐parameter, staging of the posterior corneal surface from 0 to 4; C parameter, staging of the corneal thickness from 0 to 4; inf, inferior; posterior elevation of thinnest point, elevation data of the posterior corneal surface at the thinnest point of the cornea; *K*, keratometry; *K*_max_, maximum keratometry value; *K*_mean_, mean keratometry value; sup, superior; Thinnest point thickness, minimum corneal thickness. (b) Box‐plot of the Screening Corneal Objective Risk of Ectasia (SCORE), SS‐OCT parameter as a function of the ABC classification.

Figure 3 shows the SS‐OCT parameters in relation to staging according to the ABC classification. Relevant parameters of the anterior corneal surface, such as *K*_max_, *K*_max_ − opposite *K*, inferior–superior *K*_mean_ and anterior irregularities (3 mm) are displayed in relation to parameter A (the anterior corneal surface). The parameter posterior elevation at the thinnest point refers to parameter B (posterior corneal surface), whereas the minimum corneal thickness (thinnest point thickness) is represented by parameter C. For the parameters *K*_max_, posterior elevation for the thinnest point and thinnest point thickness, a clear increase or decrease (for thinnest point thickness) with a greater degree of severity was observed. However, a slight overlap was noted in the severity levels of *K*_max_ – opposite *K*, inferior–superior *K*_mean_ and anterior irregularity (3 mm). Moreover, the SCORE parameter increased with higher severity in relation to parameters A, B and C; however, an overlap was also noted between stages (Figure 3b).

To quantify these observations, a correlation analysis was performed with the basic ABC staging parameters, that is, anterior radius of curvature, posterior radius of curvature and minimum corneal thickness (Table 3). A strong negative correlation was observed between *K*_max_ and anterior radius of curvature (*r* = −0.91, *p* < 0.001), posterior elevation at the thinnest point and posterior radius of curvature (*r* = −0.90, *p* < 0.001) and between the thinnest point thickness and minimum corneal thickness (*r* = 0.97, *p* < 0.001). There was a moderate statistically significant correlation between *K*_max_ − opposite *K*, inferior–superior *K*_mean_, anterior irregularity (3 mm) and anterior radius of curvature (all *p* < 0.001). The SCORE parameter was significantly correlated with anterior radius of curvature, posterior radius of curvature and minimum corneal thickness (*p* < 0.001).

**TABLE 3:** Correlation of swept‐source optical coherence tomography parameters with anterior and posterior radii of curvature and corneal thickness.

Parameter	ARC (mm)	PRC (mm)	MCT (mm)
Ant. *K*_max_ (D)	*r* = −0.91	–	–
	*p* < 0.001	–	–
Ant. *K*_max_ – opposite *K* (D)	*r* = −0.43	–	–
	*p* < 0.001	–	–
Ant. Inf. – sup. *K*_mean_ (D)	*r* = −0.61	–	–
	*p* < 0.001	–	–
Ant. irregularity (3 mm)	*r* = −0.435	–	–
	*p* < 0.001	–	–
Post. elev. of thinnest point (μm)	–	*r* = −0.90	–
	–	*p* < 0.001	–
Thinnest point thickness (μm)	–	–	*r* = 0.97
	–	–	*p* < 0.001
SCORE	*r* = −0.73	*r* = −0.75	*r* = −0.522
	*p* < 0.001	*p* < 0.001	*p* < 0.001

Abbreviations: Ant, anterior; ARC, anterior radius of curvature; elev, elevation; inf, inferior; *K*, keratometry; *K*_max_, maximum keratometry value; *K*_mean_, mean keratometry; MCT, minimum corneal thickness measured with RSC tomography; Post. elev. of thinnest point, elevation data of the posterior corneal surface at the thinnest corneal thickness; PRC, posterior corneal curvature; *r*, Pearson correlation; SCORE, Screening Corneal Objective Risk of Ectasia; sup, superior.

Table 4 lists the staging of the relevant parameters from the previous correlation analysis. Because of the overlap of data points between the different severity categories, for example for *K*_max_, a more robust classification was made, that is, mild, moderate and advanced (Figure 3). Figure 3 shows that around 25% of the data points in the A0 and A1 severity groups overlapped. This would result in a low discriminatory power between these two severity groups and would not allow a clear cut‐off value. In the following, the cut‐off values for these different stages were determined based on the Youden index. *K*_max_ (for the anterior corneal surface), posterior elevation for the thinnest point (for the posterior corneal surface) and thinnest point thickness (for corneal thickness) showed the best sensitivity and specificity for distinguishing between mild and moderate as well as between moderate and advanced cases. However, differentiating the various severity levels using the SCORE parameter was more difficult, which was reflected by a lower Youden index, sensitivity and specificity (Table 4).

**TABLE 4:** Staging of the swept‐source optical coherence tomography (SS‐OCT) parameters.

Parameter		Mild	Moderate	Advanced
Ant. Kmax (D)		≤50.9	51–55.9	≥56
	Youden‐Index	0.715		‐
	Sn/Sp	82%/89%		‐
	Youden‐Index	‐	0.769	
	Sn/Sp	‐	90%/87%	
Post. elev. of thinnest point (μm)		≤30	31–69	≥70
	Youden‐Index	0.626		‐
	Sn/Sp	89%/73%		‐
	Youden‐Index	‐	0.773	
	Sn/Sp	‐	83%/94%	
Thinnest point thickness (μm)		≥472 μm	471–438	≤437
	Youden‐Index	0.878		‐
	Sn/Sp	92%/96%		‐
	Youden‐Index	‐	0.796	
	Sn/Sp	‐	89%/91%	
SCORE in relation to the A‐parameter		≤17	17.1–19.6	≥19.7
	Youden‐Index	0.443		‐
	Sn/Sp	53%/92%		‐
	Youden‐Index	‐	0.464	
	Sn/Sp	‐	85%/61%	
SCORE in relation to the B‐parameter		≤8.3	8.4–19	≥19.1
	Youden‐Index	0.366		‐
	Sn/Sp	73%/63%		‐
	Youden‐Index	‐	0.684	
	Sn/Sp	‐	75%/94%	

*Note*: A‐parameter, stage of the anterior corneal radius from 0 to 4; B‐parameter, stage of the posterior corneal radius from 0 to 4; P Post. elev. of thinnest point, elevation data of the posterior corneal surface at the thinnest corneal thickness.

Abbreviations: Ant, anterior; Kmax, maximum keratometry value; SCORE, Screening Corneal Objective Risk of Ectasia; Sn, sensitivity; Sp, specificity; sup, superior.

### Diagnostic performance of tomographic parameters of the RSC and SS‐OCT

**FIGURE 4:**
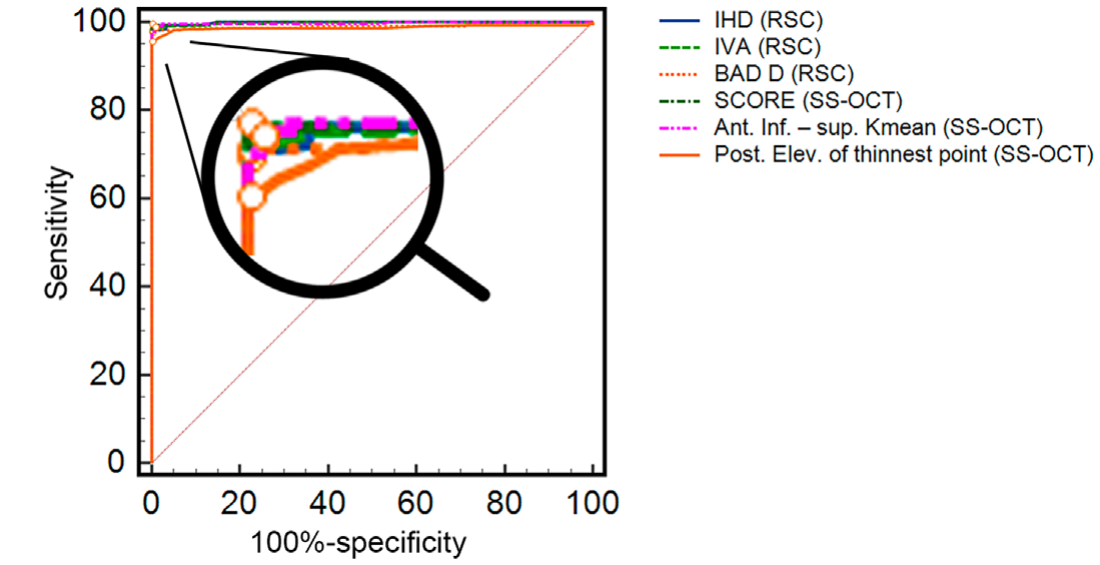
Receiver operating characteristic curves of the parameters with the highest discriminatory power between healthy and keratoconic eyes. Ant, anterior; BAD‐D, Belin/Ambrosio total value; index of height decentration, Index of Height Decentration; Inf, inferior; IHD, Index of height decentration; IVA, Index of Vertical Asymmetry; *K*_mean_, mean keratometry value; Posterior (Post) elevation (Elev) of thinnest point, elevation data of the posterior corneal surface at the thinnest point of the cornea; SCORE, Screening Corneal Objective Risk of Ectasia; SS‐OCT, swept‐source optical coherence tomography; sup, superior.

The indices of the RSC and SS‐OCT tomographs were also evaluated for diagnostic performance. ROCs (Figure 4) were created for each index and compared. All examined total corneal tomography indices achieved an AUC >0.8 (Data S3). Table 5 and Figure 4 summarise the best parameters for each device. The index of height decentration, index of vertical asymmetry and BAD‐D parameters for the RSC revealed a sensitivity/specificity of 98%/100% (cut‐off > 0.03), 99%/100% (cut‐off > 0.25) and 98%/100% (cut‐off > 2.31) in differentiating between healthy and KC eyes, respectively. The SS‐OCT parameters achieved similar results, with sensitivity/specificity of 99%/100% (cut‐off > 0.80), 99%/99% (cut‐off > 0.82) and 96%/100% (cut‐off > 16) for SCORE, inf – sup *K*‐means and posterior elevation at the thinnest point, respectively. The AUC values were significantly higher for the index of height decentration compared with the posterior elevation of the thinnest point (*p* = 0.04) and index and vertical asymmetry index compared with the posterior elevation of the thinnest point (*p* = 0.04), SCORE compared with BAD‐D and SCORE compared with the posterior elevation of thinnest point (*p* = 0.05).

**TABLE 5:** Comparison of the receiver operating characteristic curves (ROC curves) between the indices of the SS‐OCT and the RSC.

Parameter	Device	AUC	95% CI	Cut‐off	Sn	Sp	*p*‐Value^a^				
							SCORE	IVA	Ant. Inf. – sup. *K*_mean_ (D)	BAD‐D	Post. elev. of thinnest point (μm)
IHD	RSC	0.999	0.990–1.0	>0.03	0.98	1.0	0.61	0.55	0.71	0.07	**0.04**
SCORE	SS‐OCT	0.999	0.990–1.0	>0.80	0.99	1.0	–	0.19	0.55	**0.049**	**0.03**
IVA	RSC	0.998	0.989–1.0	>0.25	0.99	0.99	–	–	0.93	0.06	**0.04**
Ant. Inf. – sup. *K*_mean_ (D)	SS‐OCT	0.998	0.988–1.0	>0.82	0.99	0.99	–	–	–	0.13	0.08
BAD‐D	RSC	0.991	0.977–0.998	>2.31	0.98	1.0	–	–	–	–	0.24
Post. elev. of thinnest point (μm)	SS‐OCT	0.989	0.975–0.996	>16	0.96	1.0	–	–	–	–	–

Abbreviations: Ant, anterior; AUC, area under the curve; BAD‐D, Belin/Ambrosio total deviation value; CI, confidence intervals; elev, elevation; IHD, Index of height decentration; Inf, inferior; IVA, Index of vertical asymmetry; *K*_mean_, mean keratometry value; Post. elev. of thinnest point, elevation data of the posterior corneal surface at the thinnest corneal thickness; ROC, receiver operating characteristics; RSC, Scheimpflug‐based tomography; SCORE, Screening Corneal Objective Risk of Ectasia; Sn, Sensitivity; Sp, Specificity; SS‐OCT, Swept‐source OCT‐based tomography; sup, superior.

^a^ DeLong test, significance is marked in bold.

### Epithelial thickness measurement of the SS‐OCT

The epithelial thickness data for healthy and KC eyes are presented in Table 6. The mean epithelial thickness of the central 2‐mm zone was significantly different between healthy and KC eyes (*p* < 0.001) and between all stages of KC (all *p* < 0.05). The more advanced the KC, the thinner the epithelium in the 2‐mm zone. When the measurement zone was enlarged to 4 mm, the difference between healthy and KC eyes remained significant; however, the thinning of the epithelium was only significant between mild and advanced KC eyes (*p* = 0.002). No differences were found in the study cohort for the 6‐mm zone (all *p* > 0.05). The inferior–superior epithelium difference was significantly different between healthy and KC eyes, as well as between mild and advanced KC (*p* = 0.004), indicating a thickening effect in the upper portion to compensate for the central thinning of the epithelium in KC. The deviation of the epithelial thickness distribution was significantly higher in eyes with KC than in healthy eyes and the stage of KC was also higher (all *p* < 0.05).

**TABLE 6:** Epithelium thickness distribution of healthy and keratoconic (KC) eyes using the Swept‐source OCT‐based tomography (SS‐OCT) presented as median (IQR).

Parameter	Healthy	Keratoconus							
		Overall	*p*‐Value*	Mild	Moderate	Advanced	*p*‐Value**	*p*‐Value***	*p*‐Value****
Epithelium 2 mm zone	50.5 (48.0–53.0)	47.0 (43.0–50.0)	**<0.001**	49.0 (46.0–52.0)	47.0 (44.0–49.0)	44.0 (41.0–48.0)	**<0.001**	**<0.001**	**0.005**
Epithelium 4 mm zone	50.0 (48.0–52.0)	48.0 (46.0–51.0)	**<0.001**	49.0 (47.0–51.0)	48.0 (46.0–51.0)	47.0 (45.0–50.0)	0.16	**0.002**	0.16
Epithelium 6 mm zone	50.0 (48.0–52.0)	50.0 (48.0–52.0)	0.81	50.0 (48.0–52.0)	50.0 (47.0–52.0)	50.0 (48.0–52.25)	0.90	0.50	0.66
Epithelium I‐S	2.0 (1.0–4.0)	0.0 (−2.0–1.0)	**<0.001**	0.0 (−1.0–1.0)	0.0 (−2.0–1.0)	−1.0 (−4.0–1.25)	0.36	**0.004**	0.05
Epithelium thickness std	2.0 (2.0–3.0)	5.0 (4.0–6.0)	**<0.001**	4.0 (3.0–5.0)	5.0 (4.0–6.0)	7.0 (6.0–9.0)	**<0.001**	**<0.001**	**<0.001**

*Note*: Statistical significance is marked in bold using the Mann–Whitney *U* test.

Abbreviations: I–S, inferior–superior difference; std., standard deviation.

* *p*‐Value for healthy compared with all KC eyes.

** *p*‐Value for mild compared with moderate KC eyes.

*** *p*‐Value for mild compared with advanced KC eyes.

**** *p*‐Value for moderate to advanced KC eyes.

## DISCUSSION

Corneal tomography has been a crucial development in anterior segment imaging in recent decades. Scheimpflug‐based tomography was a pioneering development, followed by the introduction of OCT technology to perform corneal tomography with Casia 1000, Casia 2 (both from Tomey Corporation, tomey.com/tomeycorp/product.html) and MS‐39 (CSO, csoitalia.it/). ANTERION is a new anterior segment OCT technique that provides complete ocular biometry as well as corneal imaging. Biomechanical evaluation of the cornea using air impact tonometry (e.g., Ocular Response Analyser and Corvis ST) is an additional factor in ectasia screening that enables a high degree of discriminatory ability to detect early or clinical ectasia.^4^ Furthermore, the combination of biomechanical and tomographic analyses of the cornea provides detailed insights into the detection of forme fruste KC and assessment of corneal susceptibility to this disease.^23^, ^24^ New technologies, such as Brillouin microscopy, could become clinically relevant in the future.^25^

Corneal measurement using SS‐OCT tomography (ANTERION) was extended with a comprehensive software update that included an ectasia screening tool and epithelial mapping. To the best of our knowledge, this study is the first to analyse the newly introduced topographic and tomographic parameters related to KC severity. The main results were as follows: *K*_max_ and posterior elevation of the thinnest and thinnest point thicknesses were most strongly correlated with the anterior radius of curvature, the posterior radius of curvature and minimum corneal thickness on the RSC tomograph (Pentacam HR), thereby allowing the derivation of the possible staging of KC. Furthermore, the parameters SCORE, inferior–superior *K*_mean_ and posterior elevation of the thinnest point of the SS‐OCT tomograph showed high discriminatory power between healthy and KC eyes when compared with the parameters of the RSC system.

The severity of KC can vary, which affects the participant's visual performance as well as the stage‐appropriate therapy.^26^ The more severe the KC, the worse the visual acuity both with and without glasses. Rigid gas‐permeable or scleral contact lenses can more effectively improve visual performance by correcting irregular astigmatism and associated higher‐order aberrations, when compared with spectacles.^27^, ^28^

Several grading systems are available to classify KC into four stages. The most commonly used grading scheme is that of Amsler.^29^ It has subsequently been modified based on keratometry, corneal thickness, degree of astigmatism, degree of myopic refraction and slit‐lamp findings.^30^ The Collaborative Longitudinal Evaluation of Keratoconus Study (CLEK) classified KC based on steep keratometry, slit‐lamp findings and the participant's visual performance and quality.^31^ However, these classification systems do not completely reflect the tomographic characteristics of the cornea. Therefore, the Global Consensus on Keratoconus and Ectatic Diseases called for the evaluation of KC and its progression to be based on corneal tomography of the anterior and posterior surfaces and corneal thickness.^32^ The ABC classification of the RSC tomography used in this study, developed by Belin and Duncan, is based on this consideration.^20^

In the present study, the KC cohort was relatively evenly divided into stages 0, 1 and 4 according to the parameter A of the anterior corneal surface (~20%). There were a higher number of stage 2 KC eyes (35%) and a lower number of stage 3 KC eyes (9%). A less homogeneous distribution was observed for the posterior corneal surface (parameter B) than parameter A. Stages 2 (37%) and 4 (40%) were the most common, whereas eyes with stages 0 (6%), 1 (7%) and 3 (11%) were less frequent. A similar distribution of KC stages was found in the study population of the Homburg Keratoconus Centre,^33^ where a higher number of eyes were classified as A0 and A1 compared with B0 and B1 (present study: A0: 20% > B0: 6%, A1: 16% > B1: 7%) within a comparable age distribution. In contrast to the Homburg study, eyes classified as A2 (35%) or B2 (37%) were evenly distributed in the present study. Furthermore, both studies showed a lower and almost equal numbers of eyes with stages A3 and B3 (A3: 9% and B3: 11%). In stages A4 and B4, the proportions changed in the present study, with twice as many eyes classified as B4 (40%) than as A4 (20%). This was also observed in the Homburg study and led the authors to hypothesise that the ratio of the anterior to posterior corneal curvature in advanced KC changes as the posterior corneal surface becomes more curved.^33^ The distribution of corneal thickness (C parameter) stages 0–3 ranged from 20% to 30%, with only 5% of all KC eyes classified as stage 4. The Homburg study, in contrast to the present investigation, found a significant decrease in the number of eyes classified as C2, C3 and C4 compared with C0 and C1, with C4 also representing the lowest proportion of eyes.^33^

This cohort ultimately represented all stages of KC in terms of the anterior corneal surface, posterior corneal surface and corneal thickness, which is an essential requirement for subsequent staging of SS‐OCT parameters. Relevant anterior corneal surface parameters (*K*_max_, *K*_max_ − opposite *K*, inf–sup *K*_mean_ and anterior irregularity [3 mm]) were analysed in relation to the parameter A or anterior radius of curvature of the RSC tomograph. The more severe the KC, the higher the values of the individual parameters. Nevertheless, a certain overlap in the distribution of data between the analysed stages was observed for *K*_max_ − opposite *K*, inf–sup *K*_mean_ and anterior irregularity (3 mm). Consequently, the correlation coefficients of these parameters with anterior radius of curvature were between 0.43 and 0.60, which can be considered a moderate correlation. The strongest correlation was observed between *K*_max_ and anterior radius of curvature, with a correlation coefficient of 0.90, which was then used to categorise KC stages. In contrast to the stages (0–4) of the ABC classification, the statistical evaluation indicated that a more robust classification should be preferred; otherwise, there would not have been a clear separation between the stages.

The following stages were defined: mild (*K*_max_ ≤ 50.9 D), moderate (*K*_max_ between 51 and 55.9 D) and advanced (*K*_max_ ≥ 56 D). This classification achieved a clinically acceptable discriminatory ability with a sensitivity/specificity of 82%/89% between mild and moderate and 90%/87% between moderate and advanced. The 25th quartile and 75th quartile (data not shown) of the corresponding steep keratometry values for these groups were 44–47, 47–50 and 51–56 D for eyes with mild, moderate and advanced KC. These values are in strong agreement with the CLEK classification (mild, <45 D; moderate, 45–52 D; advanced, >52 D).^31^

Elevation data around the thinnest point (posterior elevation of the thinnest point) from the ectasia view were selected as the representative parameter of the posterior corneal surface on the SS‐OCT tomograph. Here, an increase in values was also observed with a higher parameter B (RSC) and a strong correlation of 0.90 with the posterior radius of curvature (RSC). Consequently, a separation was made between mild (post‐elevation of the thinnest point ≤30 μm), moderate (post‐elevation of the thinnest point between 31 and 69 μm) and advanced (post‐elevation of the thinnest point ≥70 μm). Similarly, the sensitivity/specificity was 89%/73% between mild and moderate and 83%/94% between moderate and advanced.

As anticipated, the thinnest point of the cornea on SS‐OCT decreased with the severity of the parameter C, as both parameters described the same corneal properties (*r* = 0.97 for minimum corneal thickness). Based on the classification of the anterior and posterior corneal surfaces, the stages were divided into mild (thinnest point thickness ≥ 472 μm), moderate (thinnest point thickness between 471 and 438 μm) and advanced (thinnest point thickness ≤ 437 μm), achieving sufficiently good clinical sensitivity/specificity (92%/96% between mild and moderate; 89%/91% between moderate and advanced).

The SCORE parameter was more challenging to differentiate based on KC severity. Although the correlation coefficients for the relationship between SCORE and anterior or posterior radius of curvature were 0.73 and 0.74, respectively, indicating a strong relationship, lower sensitivity or specificity was found in distinguishing between mild and moderate and between moderate and advanced. This is applied to both the anterior and posterior corneal surfaces. In addition, the cut‐off values for mild, moderate and advanced were different for A (<17, between 17.1 and 19.6 and >19.7 for mild, moderate and advanced, respectively, with respective B values of <8.3, between 8.4 and 19 and >19.1). Therefore, universal staging of KC based on the SCORE parameters alone is not recommended.

As anticipated, all analysed parameters of SS‐OCT and RSC tomography differed significantly between healthy eyes and those with KC. However, this did not qualify the parameters or indices directly as screening or diagnostic parameters. Accordingly, further analysis was performed to assess the discriminative ability of healthy controls and KC participants. All corneal tomography parameters achieved an AUC value >0.80. If the parameters allowed for optimal separation between the two cohorts without overlapping data points, the AUC would be 1.0. The parameters with the highest discriminatory power (AUC > 0.98) were SCORE, inferior–superior *K*_mean_ and posterior elevation of the thinnest point for SS‐OCT and the index of height decentration, index of vertical asymmetry and BAD‐D for RSC. Topography (inferior–superior *K*_mean_, index of height decentration and index of vertical asymmetry) and tomography (SCORE, posterior elevation of the thinnest point and BAD‐D) parameters of SS‐OCT and RSC were presented.

In comparison with previous studies, the RSC tomography parameters index of height decentration, index of vertical asymmetry and BAD‐D had a higher discriminatory ability in this study, which was also reflected by their high sensitivity and specificity. The index of height decentration, which achieved an AUC value of 0.999 with a sensitivity/specificity of 98%/100%, had previously shown non‐homogeneous results. Shetty et al. found a sensitivity of 100% but only a specificity of 45.7% (AUC 0.99) with a cut‐off of 0.02, which is lower than that in the present study (0.03).^15^ However, higher sensitivities and specificities have been reported in other studies.^11^, ^34^ The results of this study were consistent with those of Tian et al. and Heidari et al. in terms of AUC.^13^, ^34^ The index of height decentration is the vertical decentration of the height data of a 3‐mm ring based on Fourier analysis, with a value >0.02 being pathological according to the instrument manufacturer.^35^ Using the horizontal meridian as the axis of reflection, the index of vertical asymmetry describes the mean difference between the superior and inferior corneal curvatures. Values >0.32 are considered pathological.^35^ In this study, a cut‐off value of 0.25 was found, which showed high sensitivity, specificity and AUC values of 99%, 99% and 0.998, respectively. In previous studies, AUC values ranged from 0.90 to 0.997.^11^, ^12^, ^13^, ^14^, ^15^, ^34^ The consistently favourable results make this index a valuable parameter for KC assessment. A tomographically based parameter, the BAD‐D, is one of the three most crucial parameters of an RSC tomograph in terms of discriminatory power. It comprises information on the elevation of the anterior corneal surface, posterior corneal surface and pachymetric data based on the thinnest part of the cornea.^36^, ^37^ In similar study populations, this parameter achieved AUC values between 0.972 and 0.999,^10^, ^13^, ^15^ with a sensitivity/specificity of 97%/95%.^10^ Hashemi et al.^10^ reported results comparable to the present work with a similar cut‐off value of 2.38 (here: 2.31).

The small differences between the studies were probably due to the inclusion and exclusion criteria. The present study used objective criteria, in addition to clinical findings. Moreover, the differences in results may be due to the composition of the cohorts, depending on the number of mild, moderate and advanced cases. In the present study, more than half (65%) of the participants were classified as having moderate or advanced disease. Consequently, the parameters achieved a high level of distinctiveness.

The SS‐OCT parameter with the highest discrimination was the inferior–superior *K*_mean_, the posterior elevation of the thinnest point and a combination of topographic and tomographic data (SCORE). The parameter inferior–superior *K*_mean_ is, by definition, comparable to the index of vertical asymmetry parameter of the RSC tomography, which was also reflected in the sensitivity (99%), specificity (99%) and AUC results (0.998). The parameters of the epithelium currently displayed in the software did not provide a higher level of discrimination between healthy and KC eyes compared with other tomographic data. Although there were significant differences in some parameters between the two cohorts, the AUC values only ranged from 0.51 to 0.92, with the best parameter, i.e., the standard deviation of epithelial thickness, with a high sensitivity/specificity of 80%/91%. Given the high variability in epithelial thickness distribution, it should be analysed in specific regions or sectors rather than in zones or mean values.^38^ Differences between these regions or sectors may also be more appropriate for ectasia screening.^39^

The SCORE parameter, which combines topographic and tomographic data into an overall index, was comparable to the BAD‐D parameter of the RSC tomograph. However, they differed in terms of their machine learning algorithms, training and validation in the development process and the parameters used (topography and tomography). The SCORE parameter achieved a statistically significantly higher AUC than BAD‐D; however, this cannot be considered clinically relevant, as the sensitivity (99%) and specificity (100%) were clinically similar to the BAD‐D (98%/100%).

As previously mentioned, SCORE was less effective for classifying KC in this study. However, it demonstrated high accuracy in detecting manifest KC as it was designed as a screening index based on discriminant analysis, as used by Saad et al.^19^ The machine learning algorithm was primarily used to differentiate between healthy, forme fruste KC and clinical KC, and not directly to classify KC, which is shown graphically in Figure 3b. There was a favourable separation between the healthy and A0/A1, B0/B1 and C0/C1 groups. To avoid incorrect clinical or scientific conclusions, it is essential to be clear about the training methods and intended purposes of the analysis.^40^ The current study did not include eyes with forme fruste KC or cases of very asymmetric ectasia, indicating that no conclusions can be drawn regarding the usefulness of SCORE in detecting early corneal ectasia, which is a limitation of this investigation. This issue will be addressed in future studies.

A further limitation of the present study is that the proposed staging was based on data from one study centre, and validation in other centres is essential. In addition, the classification cannot be used directly for progression analysis, which is a disadvantage compared with the ABCD classification.^41^ However, the results of the repeatability studies conducted to date could be used to differentiate clinical changes from the measurement noise of SS‐OCT.^16^, ^17^ In a previous investigation, the most commonly used clinical parameter, *K*_max_, showed a coefficient of repeatability of 0.7 D for eyes with moderate KC, well below the 1 D limit. Therefore, an increase in *K*_max_ of >0.8 D may indicate a clinically significant change.^17^

## CONCLUSION

The individual parameters of the new ectasia tool and the overall parameter SCORE of the SS‐OCT differed between healthy and KC eyes. The parameters SCORE, inferior–superior *K*_mean_ and the posterior elevation of the thinnest point were comparable to those of RSC tomography in terms of diagnostic ability. Mild KC eyes can be characterised using SS‐OCT as follows: *K*_max_ ≤ 50.9 D, posterior elevation of the thinnest point ≤30 μm and thinnest point thickness ≥ 472 μm. Eyes with moderate KC had values between 51 and 55.9 D, 31 and 69 μm and 471 and –438 μm for *K*_max_, the posterior elevation of the thinnest point and thinnest point thicknesses, respectively. All participants with advanced disease were *K*_max_ ≥56 D, posterior elevation of the thinnest point ≥70 μm and thinnest point thickness ≤ 437 μm.

## AUTHOR CONTRIBUTIONS

**Robert Herber:** Conceptualization (lead); data curation (lead); formal analysis (lead); investigation (lead); methodology (lead); project administration (lead); visualization (lead); writing – original draft (lead). **Janine Lenk:** Methodology (supporting); validation (supporting); writing – review and editing (supporting). **Lisa Ramm:** Investigation (supporting); validation (supporting); writing – review and editing (equal). **Dierk Wittig:** Conceptualization (supporting); investigation (supporting); methodology (supporting). **Maria Magdalena Patzner:** Data curation (supporting); investigation (equal). **Lutz E. Pillunat:** Supervision (supporting). **Frederik Raiskup:** Conceptualization (supporting); supervision (lead); validation (lead); writing – review and editing (equal).

## FUNDING INFORMATION

No funding was available.

## CONFLICT OF INTEREST STATEMENT

All authors declare no conflicts of interest. RH has received fees as a speaker for Heidelberg Engineering GmbH.

## Supporting information


Data S1.

Data S2.

Data S3.
