# Supplementary material for: Evaluation of indices for the assessment and classification of keratoconus based on optical coherence tomography and Scheimpflug technology
Source: Ophthalmic Physiol Opt. 2024 Dec 5;45(2):391–404. doi: 10.1111/opo.13425 (PMC11823291; doi:10.1111/opo.13425)
Supplement: Supplementary file 1 — Data S1. [file OPO-45-391-s003.docx]

Supplement 1 Bland-Altman analysis of anterior and posterior corneal parameters between RSC and SS-OCT.

|  |  | **Healthy** | **P-value** | **Beta** | **P-value** | **Keratoconus** | **P-value** | **Beta** | **P-value** |
| --- | --- | --- | --- | --- | --- | --- | --- | --- | --- |
|  |  | offset (lower LoA - upper LoA) |  |  |  | offset (lower LoA - upper LoA) |  |  |  |
| anterior | K steep (D) | -0.27 (-0.74 - 0.2) | **< 0.001** | -0.004 | 0.803 | -0.13 (-1.78 - 1.52) | **0.004** | 0.070 | **< 0.001** |
|  | CA (D) | -0.03 (-0.37 - 0.31) | 0.066 | -0.002 | 0.858 | -0.35 (-2.23 - 1.53) | **< 0.001** | -0.06 | **0.045** |
|  | Kmax (D) | -0.19 (-0.86 - 0.48) | **< 0.001** | 0.002 | 0.926 | 0.86 (-2.23 - 3.94) | **< 0.001** | 0.104 | **< 0.001** |
| posterior | BFS (mm) | -0.15 (-0.26 – (-0.05)) | **<0.001** | 0.036 | 0.108 | -0.09 (-0.35 – 0.17) | **< 0.001** | 0.005 | 0.802 |
|  | Elevation at thinnest point (µm) | 0.7 (-5.71 - 7.12) | **0.022** | 0.02 | 0.830 | -13.28 (-43.04 - 16.5) | **<0.001** | -0.341 | **< 0.001** |
|  | Thinnest point thickness (µm) | 0.15 (-11.21 - 11.5) | 0.787 | -0.02 | 0.257 | -1.37 (-22.11 - 19.37) | **0.018** | -0.023 | 0.106 |

Beta, slope of the linear regression formula; BFS, best-fit sphere; CA, corneal astigmatism; LoA, limits of agreement; RSC, rotating Scheimpflug camera; K, keratometry value; Kmax, maximum keratometry, SS-OCT, swept-source optical coherence tomography. Significance is marked in bold.
