# Supplementary figures and images for: Evaluation of indices for the assessment and classification of keratoconus based on optical coherence tomography and Scheimpflug technology
Source: Ophthalmic Physiol Opt. 2024 Dec 5;45(2):391–404. doi: 10.1111/opo.13425 (PMC11823291; doi:10.1111/opo.13425)

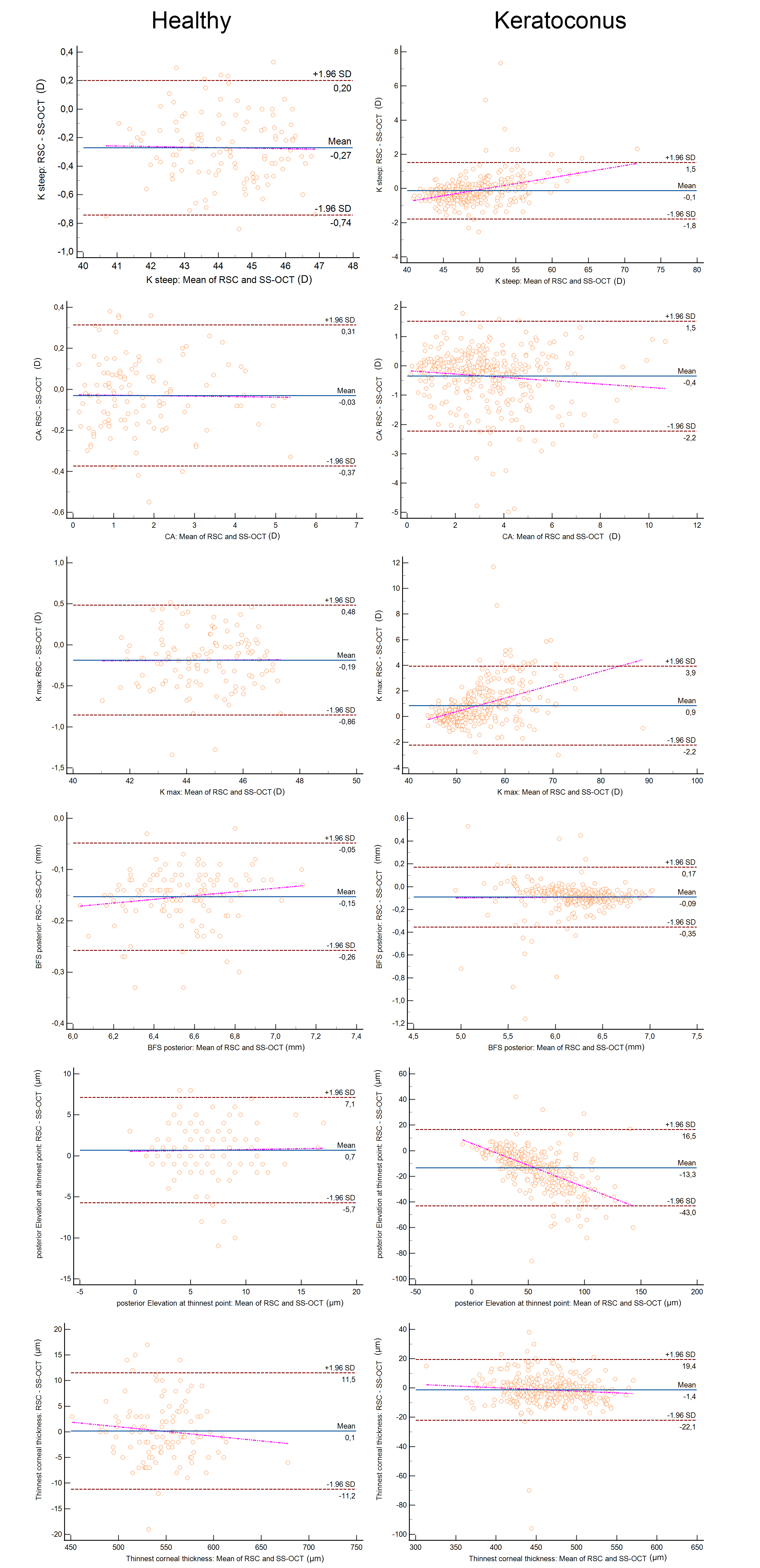

Supplement: Supplementary file 2 — Data S2. [file OPO-45-391-s002.tif]
