# Supplementary material for: Evaluation of indices for the assessment and classification of keratoconus based on optical coherence tomography and Scheimpflug technology
Source: Ophthalmic Physiol Opt. 2024 Dec 5;45(2):391–404. doi: 10.1111/opo.13425 (PMC11823291; doi:10.1111/opo.13425)
Supplement: Supplementary file 3 — Data S3. [file OPO-45-391-s001.docx]

Supplement 3 Comparison of the receiver operating characteristic curves (ROC curves) between all indices of the SS-OCT and the RSC.

| Parameter | Device | AUC | 95%-CI | Cut-off | Sn | Sp |
| --- | --- | --- | --- | --- | --- | --- |
| IHD | RSC | 0.999 | 0.990 - 1.0 | > 0.032 | 0.98 | 1.0 |
| IVA |  | 0.998 | 0.989 - 1.0 | > 0.25 | 0.99 | 0.99 |
| KI |  | 0.996 | 0.985 - 1.000 | > 1.05 | 0.98 | 0.99 |
| BAD D |  | 0.991 | 0.977 - 0.998 | > 2.31 | 0.98 | 1.0 |
| KISA |  | 0.989 | 0.975 - 0.997 | > 26.2 | 0.95 | 0.97 |
| ISV |  | 0.988 | 0.973 - 0.996 | > 38 | 0.91 | 0.97 |
| Ele B Thinnest point (µm) |  | 0.987 | 0.972 - 0.995 | > 19 | 0.94 | 1.0 |
| ART Max. |  | 0.985 | 0.969 - 0.994 | ≤ 330 | 0.97 | 0.99 |
| K max (D) |  | 0.984 | 0.968 - 0.994 | > 47 | 0.93 | 1.0 |
| RPI Avg. |  | 0.980 | 0.962 - 0.991 | > 1.24 | 0.93 | 1.0 |
| MCT (µm) |  | 0.944 | 0.919 - 0.963 | ≤ 509 | 0.85 | 0.9 |
| IHA |  | 0.895 | 0.862 - 0.921 | > 15.9 | 0.70 | 0.93 |
| CKI |  | 0.806 | 0.766 - 0.841 | > 1.01 | 0.74 | 0.98 |
| SCORE | SS-OCT | 0.999 | 0.990 - 1.0 | > 0.8 | 0.99 | 1.0 |
| Ant. Inf. – sup. Kmean (D) |  | 0.998 | 0.988 - 1.0 | > 0.82 | 0.99 | 0.99 |
| Post. elev. of thinnest point (µm) |  | 0.989 | 0.975 - 0.996 | > 16 | 0.96 | 1.0 |
| Ant. KMax (D) |  | 0.980 | 0.962 - 0.991 | > 47.2 | 92.8 | 98.3 |
| Ant. irregularity (3mm) |  | 0.965 | 0.943 - 0.980 | > 1.5 | 93.1 | 83.8 |
| Ant. KMax - opposite K (D) |  | 0.951 | 0.927 - 0.969 | > 2.5 | 85.4 | 99.1 |
| Thinnest point thickness (µm) |  | 0.937 | 0.910 – 0.957 | ≤ 508 | 84.2 | 88.0 |
| Epithelium thickness std | SS-OCT | 0.921 | 0.890 – 0.946 | > 3 | 80.1 | 91.2 |
| Epithelium I-S |  | 0.814 | 0.772 – 0.852 | ≤ 0 | 61.7 | 90.2 |
| Epithelium 2 mm zone |  | 0.729 | 0.682 – 0.773 | ≤ 48 | 63.1 | 73.5 |
| Epithelium 4 mm zone |  | 0.648 | 0.598 – 0.696 | ≤ 49 | 65.5 | 56.9 |
| Epithelium 6 mm zone |  | 0.508 | 0.456 – 0.560 | ≤ 54 | 94.5 | 12.0 |

Ant, anterior; ART max, Ambrosio Relationed Thickness; BAD D, Belin/Ambrosio total deviation value; CI, confidence interval; CKI, Center Keratoconus Index; Ele B BFS 8mm Thinnest, elevation data of the posterior corneal surface at the thinnest corneal thickness; K, keratometry; KI, Keratoconus Index; K max, maximum keratometry value; Kmean, mean keratometry value; IHA, Index of Height Asymmetry; IHD, Index of Height Decentration; inf, inferior; ISV, Index of Surface Variance; IVA, Index of Vertical Asymmetry; I-S, inferior-superior difference; MCT, minimal corneal thickness; Post. elev. of thinnest point, elevation data of the posterior corneal surface at the thinnest corneal thickness; RPI Avg, averaged pachymetric progression; SCORE, Screening Corneal Objective Risk of Ectasia; std, standard deviation; sup, superior.
